# Novel application of drug-coated balloons in coronary heart disease: A narrative review

**DOI:** 10.3389/fcvm.2023.1055274

**Published:** 2023-03-02

**Authors:** Lijin Wang, Xiaokang Li, Tian Li, Lin Liu, Haiyan Wang, Chiyao Wang

**Affiliations:** ^1^Department of Cardiology, Tangdu Hospital, Fourth Military Medical University, Xi'an, China; ^2^School of Basic Medicine, Fourth Military Medical University, Xi'an, China; ^3^Department of Dermatology, Tangdu Hospital, Fourth Military Medical University, Xi'an, China; ^4^Department of Structural Heart Disease, The First Affiliated Hospital of Xi'an Jiaotong University, Xi'an, China

**Keywords:** coronary heart disease, drug-coated balloon, drug-eluting stent, percutaneous transluminal coronary intervention, CAD

## Abstract

The incidence of coronary heart disease (CAD) has soared over the years, and coronary intervention has become an increasingly important therapeutic approach. The past decade has witnessed unprecedented developments in therapeutic medical instruments. Given that drug-coated balloons bring many benefits, they are indicated for an increasing number of conditions. In this article, we review the results of current clinical trials about drug-coated balloons and summarize their safety and clinical progression in different coronary artery diseases, laying the groundwork for basic research, and clinical therapeutics of this patient population.

## 1. Introduction

Current evidence suggests that the incidence of coronary heart disease has increased in recent years. By the end of 2016, there were about 126 million patients with coronary heart disease worldwide. Percutaneous coronary intervention (PCI) remains the mainstay of treatment in this patient population (1, 2). The application of bare metal stents (BMS) has significantly improved the prognosis of patients undergoing interventional therapy but brings a high risk of in-stent restenosis (ISR) and in-stent thrombosis (3). Subsequently, drug-eluting stents (DES) delivering antiproliferative drugs have been developed and reported to reduce the incidence of restenosis dramatically from 30% to about 5%, becoming the main method of coronary intervention therapy (4). However, many complications are associated with the stent itself, such as late stent thrombosis, long-term antiplatelet therapy, in-stent restenosis and so on (5). For this reason, the concept of “intervention without implantation” emerged early this century, and drug-coated balloons (DCB) came into being.

**Figure 1:**
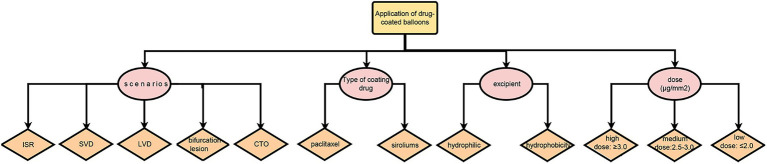
Application of drug-coated balloons.

Drug-coated balloons are special interventional devices with antiproliferative drugs on the balloon’s surface (Figure 1). During the procedure, the balloon reaches the lesion site through the catheter and is dilated for 30–60s. Meantime, drugs coated on the balloon’s surface are evenly released and spread into the cells, yielding a long-lasting anti-proliferation effect on endothelial and smooth muscle cells (6–9).

Different kinds of antiproliferative drugs can be coated on the balloon or stents. Taxanes are the first documented and most common class of coated drugs, among which paclitaxel is the most predominantly used clinically. It is well-established that paclitaxel prevents cell mitosis and mainly acts as an antiproliferative drug at low concentrations. It is highly lipophilic, thus being easily absorbed by cells through lipid cell membranes, and produces a longer-lasting effect since parts of the drug remain on the cell membrane. Limus drugs represent another common category of drugs coated on balloons, including sirolimus, everolimus or tacrolimus and so on. Sirolimus, a macrocyclic lactone, is the most widely used drug in the limus family, with the ability to inhibit the mammalian target of rapamycin (mTOR) to restrict the migration and proliferation of SMCs (10). Even though sirolimus-coated balloons (SCB) and paclitaxel-coated balloons (PCB) found that the SCB and PCB yielded comparable angiographic outcomes in ISR (11, 12), different drugs coated on the balloon are suitable for different conditions, warranting further research.

Compared with DES, DCB shows many advantages: (1) there is no foreign body implanted in the patient’s body during the DCB angioplasty, which avoids changes in the original anatomical structure of the coronary artery and complications such as late stent thrombosis and allergy. Moreover, it provides opportunities for subsequent interventional therapy or other surgical treatment, especially for young patients; (2) the interventional procedure is relatively simple with a shorter operation duration and reduced radiation exposure to the medical staff and patients; (3) DCB can evenly deliver the drug to the inner wall of the blood vessel, which avoids delayed endothelialization caused by the uneven distribution of stent metal rods; (4) the application of DCB can shorten the time of dual antiplatelet therapy and reduce the risk of bleeding and other complications; (5) DCB may be a more appropriate than stent implantation for special types of lesions, such as narrow lesions, high bleeding risk lesions or the patient with diabetes, etc. (13–17).

The advantages of DCB in PCI have been confirmed in many clinical trials. DCB is reportedly the preferred choice for ISR or small vessel disease (SVD) (18). However, the clinical efficacy of DCB in coronary de-novo stenosis warrants more exploration. This article reviews the latest evidence on DCB in de-novo stenosis in coronary arteries.

## 2. Application of DCB in in-stent restenosis

In-stent restenosis is widely acknowledged as a major complication after PCI. Even though using DES decreases the incidence of ISR from 30 to 5% compared with BMS, the incidence remains high for patients with inflammation or other comorbidities such as diabetes (19, 20). Indeed, DCB was originally developed to prevent ISR (16).

A large number of clinical trials have established the efficacy of DCB in the treatment of ISR. In this respect, PACCOCATHISR-I and PACCOCATHISR-II were the first clinical trials to assess DCB in treating ISR, showing that DCB coated with paclitaxel significantly decreased the late lumen loss and diameter stenosis of the coronary artery lesions than conventional balloon angioplasty (13, 15, 21). Subsequently, several clinical trials have been conducted to observe the efficacy of DCB in ISR and yielded relatively positive results (22). For example, the ISAR DESIRE3 trial compared the efficacy of DCB coated with paclitaxel with DES eluting paclitaxel and plain balloon angioplasty on in-stent restenosis. The follow-up angiography results showed equal efficacy between DCB and DES for solving diameter stenosis, and both treatments yielded superior outcomes than plain balloon angioplasty (23). Other clinical trials also supported these results, including PEPCAD-DES (24) and PEPCAD CHINA ISR (25). Several meta-analyses summarized the results of clinical trials comparing the efficacy of balloons and stents and further confirmed that DCB and DES are equally effective in solving in-stent restenosis (26–28).

The PEPCAD-II study compared the efficacy of DCB and DES, which were coated with paclitaxel. At 6-month follow-up, the in-segment late lumen loss in the DES group was more significant than in the DEB group (0.38 ± 0.61 mm vs. 0.17 ± 0.42 mm, *p* = 0.03), while for other indicators, including MACE, TLR et al., the DCB group yielded better results (29). Consistent findings were reported by Scheler et al. (30). A meta-analysis of 27 randomized trials, including 5,923 ISR patients, analyzed the percent diameter stenosis 6–12 months after intervention by stents or balloons with different coated drugs. DCB was suggested as one of the two strategies for treating any coronary ISR based on evidence of its efficacy without adding a new stent layer (22). Another recently published meta-analysis illustrated similar results (26). Overall, DCB in patients with ISR is reportedly equally effective or even better than other kinds of DES. Randomized controlled trials of DCBs in ISR are displayed in Table 1.

**Table 1:** Clinical Trials of DCBs in ISR.

Study name	Country and time	Research design	Follow-up duration	Primary endpoint	*p*-value	Secondary endpoint	*p*-value
**BMS ISR**							
TIS (31)	Czech Republic 2016	PCB (*n* = 68) vs. EES (*n* = 68)	12 months	LLL: 0.02 mm vs. 0.19 mm	0.0004	MACE: 10.29% vs. 19.12% binary restenosis: 8.7% vs. 19.12%	0.213 0.078
RIBS V (32)	Spanish 2012	PCB (*n* = 95) vs. EES (*n* = 94)	6–9 months 12 months	LLL: 0.14 ± 0.5 mm vs. 0.04 ± 0.5 mm; binary restenosis: 9.5% vs. 4.7%	0.22	MACE: 8% vs. 6%	0.60
SEDUCE (33)	Belgium 2010	PCB (*n* = 25) vs. EES (*n* = 25)	9 months	LLL: 0.28 mm vs. 0.07 mm	0.1	–	–
PACOCATH ISR II (34, 35)	Germany 2006	PCB (*n* = 54) vs. POBA (*n* = 54)	6 months 24 months 60 months	LLL: 0.11 ± 0.45 mm vs.0.81 ± 0.79 mm	0.001	MACE: 11% vs. 46%; MACE: 27.8% vs. 59.3%	0.001 0.009
PEPCAD II (30)	Germany 2006	PCB (*n* = 66) vs. PES (*n* = 65)	6 months 12 months	LLL: 0.17 ± 0.42 mm vs. 0.38 ± 0.61 mm	0.03	BR%: 7% vs. 20% MACE: 9% vs. 22%	0. 06 0.08
PACOCATH ISR I (21)	Germany 2005	PCB (*n* = 26) vs. POBA (*n* = 26)	6 months 12 months	LLL: 0.03 ± 0.48 mm vs.0.74 ± 0.86 mm	0.002	MACE: 4% vs. 31%	0.01
**DES ISR**							
RESTORE (36)	Korea 2017	DCB (*n* = 86) vs. EES (*n* = 86)	9 months 12 months	LLL: 0.15 ± 0.49 mm vs. 0.19 ± 0.41 mm	0.54	In-segment MLD: 1.80 ± 0.69 mm vs. 2.09 ± 0.46 mm In-stent MLD: 1.90 ± 0.71 mm vs. 2.29 ± 0.48 mm In-segment DS%: 34% ± 21% vs. 26% ± 15% In-stent DS%: 33% ± 21% vs. 21% ± 15% MACE: 7.0% vs. 4.7%	0.03 0.005 0.05 0.002 0.51
ISAR DESIRE IV (37)	Germany 2016	PCB (*n* = 127) vs. SCB + PCB (*n* = 125)	6–8 months 12 months	In-segment DS%: 35.0 ± 16.8% vs. 40.4 ± 21.4%	0.047	– TLR: 21.8% vs. 16.2% MACE: 23.3% vs. 18.4%	– 0.26 0.35
PEPCAD CHINAISR (25)	China 2013	PCB (*n* = 110) vs. PES (*n* = 110)	9 months 12 months	LLL: 0.46 ± 0.51 mm vs. 0.55 ± 0.61 mm	0.0005	TLR: 14.5% vs. 13.6%	0. 84
ISAR-DESIRE 3 (23))	Germany 2012	PCB (*n* = 137) vs. PES (*n* = 131) vs. POBA (*n* = 134)	9 months	Binary restenosis: 38% vs. 37.4% vs. 54.1%	0.007	TLR: 22.1% vs. 13.5% vs. 43.5%	*P*_(PCB vs. PES)_ = 0.09，*P*_(PCB vs. POBA)_ <0.0001, *P*_(PES vs. POBA)_ <0. 0001
RIBS IV (38, 39)	Spanish 2012	DCB (*n* = 154) vs. EES (*n* = 155)	6–9 months 12 months 36 months	MLD: 1.80 ± 0.6 mm vs. 2.03 ± 0.7 mm	<0.01	MACE: 18% vs. 10% MACE: 12.3% vs. 20.1%	0.04 0.04
PEPCAD-DES (24, 40)	Germany 2011	PCB (*n* = 72) vs. POBA (*n* = 38)	6 months 36 months	LLL: 0.43 ± 0.61 mm vs. 1.03 ± 0.77 mm	< 0.001	MACE: 16.7% vs. 50.0% MACE: 20.8% vs. 52.6%	< 0.001 0.001
**Mixed ISR**							
DARE (41)	The Netherlands 2018	DCB (*n* = 141)vs. DES(*n* = 137)	6 months 12 months	MLD: 1.71 ± 0.51 mm vs. 1.74 ± 0.61 mm	<0.0001	TVR: 7.1% vs. 8.8%	0.65
BIOLUX (28)	Germany 2018	DCB (*n* = 157) vs. DES (*n* = 72)	6 months 12 months	LLL: 0.03 ± 0.40 mm vs. 0.20 ± 0.70 mm	0.40	TLF: 16.7% vs. 14.2%	0.65
DELUX registry (42)	Germany 2014	DCB (*n* = 1,064)	1 months 6 months 12 months	MACE: 8.5%	–	MACE:15.1%	–
SeQuent Please World Wide registry (43)	Germany 2012	DCB (*n* = 390)	9 months	TLR: 1.0%	–	MACE: 2.6%	–

DCB, drug-coated balloon; BMS, bare-metal stent; ISR, in-stent restenosis; DES, drug-eluting stent; EES, everolimus-eluting stent; PES, paclitaxel-eluting stent; PCB, paclitaxel-coated balloon; SCB, sirolimus-coated balloon; TLF, target lesion failure; TLR, target lesion revascularization; TVR, target vessel revascularization; POBA, plain old balloon angioplasty; MLD, minimum lumen diameter; MACE, major advance cardiovascular event; LLL, late lumen loss; DS%, percent diameter stenosis; BR%, Binary Restenosis Rate “–”: no data.

Current evidence suggests that more emphasis should be placed on drug selection for DCB. Interestingly, clinical trials found that the difference between drugs coated on the balloon could affect the clinical outcomes; the clinical trial by Carlo et al. revealed that the SCB group was associated with a lower target lesion failure rate than PCB in patients with DES ISR (12). Some studies also found that SCB yielded better results in study endpoints (11). Although the differences were not statistically different, this finding still reminds us that the application of SCB in ISR warrants strong evidence with well-balanced, adequately powered studies that will have to match the results of DES. If the initial promising results of sirolimus are confirmed, the era of a new metal layer will come.

Overall, DCB should be indicated for patients with ISR. Based on evidence from clinical trials, DCB therapy is recommended for ISR according to the 2018 ESC/EACTS myocardial revascularization guidelines (level of evidence: IA) (44).

## 3. Application of DCB in small vessel disease

Coronary artery SVD accounts for 30% of patients needing interventional therapy and is more common in women or the elderly and patients with diabetes or kidney disease (45). The small vessel diameter makes it challenging for stent implantation (46). Moreover, previous studies have shown that small vessel diameter is the strongest inducer of restenosis among all clinical factors (47), which leads to the high probability of ISR in SVD after DES treatment. Therefore, it is essential to inhibit restenosis during the treatment of SVD (48).

Under such conditions, DCB does not involve the insertion of a foreign body (49), which can greatly reduce intravascular inflammatory reaction and the probability of restenosis. Up to now, there have been several clinical randomized controlled trials on the efficacy of DCB in treating SVD. Clinical trials that assessed the use of DCBs in SVD are shown in Table 2. PEPCAD I is the first clinical study to evaluate the efficacy of DCB in small vessel lesions. In this trial, all patients were treated with DCB, of which 32 underwent emergency stent implantation. The average segmental late lumen loss (LLL) was analyzed 6 months after the operation. The follow-up results showed that the LLL of patients that underwent DCB angioplasty were superior to those treated with stents (0.16 ± 0.38 mm vs. 0.28 ± 0.53 mm), indicating that DCB alone significantly reduced the restenosis rate in small vessel lesions (52). Basket-Small 2 is the largest multicenter clinical trial comparing the efficacy of DCB and DES in SVD with level 1a evidence based on OCEBM-2011 standards, including 758 patients with small vessel lesions (diameter < 3 mm) (57). Major adverse cardiovascular events (MACE, such as cardiac death, nonfatal myocardial infarction and target vascular revascularization) were assessed in patients during 3-year follow-up, and the results showed that the incidence of MACE in both groups was equal (15%). However, the rate of vessel or stent thrombosis and severe hemorrhage in the DCB group was lower than in the DES group (58, 59), and the advantages of DCB in patients with diabetes were more significant (60–62). The BELLO trial assessed the non-inferiority of DCB vs. DES with paclitaxel which included 182 SVD patients in 2012. During the 6-month and 3-year follow-up, the incidence of target lesion revascularization (TLR) (4.4% vs.7.6%, *p* = 0.37) and MACE (10% vs.16.3%, *p* = 0.21) in the DCB group was lower than in the DES group. The study showed that DCB yielded a better therapeutic effect than DES in SVD (53). The RESTORESVD China is a clinical trial of DCB treatment in SVD conducted in China that compared paclitaxel-coated balloons with a new generation DES: the zotarolimus-eluting stent. It was found that the rates of 9-month segmental diameter stenosis and 1-year target lesion failure (TLF) were comparable between stent and balloon groups, indicating that the efficacy of DCB is not inferior to new generation DES (51). Moreover, the PICCOLETOII trial assessed 232 SVD patients from five centers for a comparative analysis of DCB and DES with everolimus and found that the LLL in the DCB group was superior to the DES group after an average follow-up of 180 days (0.04 vs. 0.17 mm; *P*_non-inferior_ < 0.001; *P*_superior-efficacy_ = 0.03). The 12-month clinical follow-up results showed that the rate of MACE in the DCB group was lower than in the DES group (5.6% vs. 7.5%*, p = 0.55*) (50). However, in some studies, such as the PICCOLETTO study, DCB failed to show equivalence to DES regarding angiographic endpoints during PCI. The PICCOLETTO study was terminated prematurely because of the high incidence of MACE in the DCB group, which may be due to inadequate preparation for balloon pre-dilatation and the design defects of the Dior I balloon used in this study.

**Table 2:** Clinical trials of DCBs in SVD.

**Study name**	**Country and time**	**Research design**	**Follow-up duration**	**Primary endpoint**	***p*-value**	**Secondary endpoint**	***p*-value**
PICCOLETO II (50)	Italy 2020	DCB (*n* = 114) vs. EES (*n* = 118)	6 months 12 months	LLL: 0.04 vs. 0.17 mm	0.001	MACE: 5.6% vs. 7.5%	0.55
BASKET-SMALL 2(38, 39)	Switzerland 2018	DCB (*n* = 382) vs. DES (*n* = 376)	12 months 36 months	MACE: 7.5% vs. 7.3%	0.9180	MACE: 15% vs. 15%	0.95
The RESTORE SVD China (51)	China 2018	PCB (*n* = 116) vs. DES (*n* = 114)	9–12 months 12 months	In-stent DS%: 29.6 ± 2.0% vs. 24.1 ± 2.0%	< 0.001	TLF: 4.4% vs. 2.6%	0.72
PEPCAD I (52)	Germany 2015	DCB (*n* = 118)	6 months	LLL: 0.16 ± 0.38 mm	–	–	–
BELLO (53)	Italy 2012	PEB (*n* = 90) vs. PES (*n* = 92)	6 months 36 months	LLL: 0.08 ± 0.38 mm vs. 0.29 ± 0.44 mm	0.001	Restenosis rate: 10.0% vs. 14.6% TLR: 4.4% vs. 7.6% MACE: 10.0% vs. 16.3% MACE: 14.4% vs. 30.4%	0.35 0.37 0.21 0.015
SeQuent SVD registry (54)	Germany 2012	DCB-only (*n* = 420) vs. DCB + BMS (*n* = 27)	9 months	TLR:3.6%vs.4.0%	0.922	MACE: 4.7% vs. 4.0%	0.866
PICCOLETO (55, 56)	Italy 2010	PCB (*n* = 28) vs. DES (*n* = 29)	6 months 9 months	DS%: 43.6% vs. 24.3%	0.02	MACE: 35.7% vs. 13.8%	0.054

Other abbreviations as in Table 1.

Overall, mounting evidence suggests that DCB is safe and feasible in treating small vascular lesions and can significantly reduce the incidence of restenosis and yield equivalent or better clinical effects than stents.

## 4. Application of DCB in large vessel disease

It is widely acknowledged that large vessels supply blood to broader areas, and lesions associated with these vessels significantly impact patient health. It has long been thought that coronary arteries have more smooth muscle fibers. Accordingly, they are more likely to exhibit elastic retraction or dissection, which may cause acute vascular occlusion. Therefore, DES providing more support to vessel walls represents the best choice for treating large vessel disease (LVD). However, recent studies have shown that DCB is feasible in macrovascular lesions, highlighting that it is safe and effective for treating LVD. A clinical trial by Yu et al. included 527 patients with 595 lesions treated with DCB, of which 222 lesions were classified in the large vessel group (diameter > 2.8 mm) and 373 lesions in the small vessel group (diameter ≤ 2.8 mm). During an average clinical follow-up of 10.1 months, the large vessel group experienced a lower incidence of MACE (0% vs. 1.4%) and target lesion revascularization (0% vs. 1.1%) than the small vessel group, and no death was observed in both groups. These results substantiated that DCB yielded good efficacy for treating large vessel disease in terms of clinical outcomes and angiographic appearance (63). It was also found that DCB yielded better effects than DES in macrovascular lesions. A study by Shin et al. compared the efficacy of DCB and DES in treating macrovascular lesions (diameter between 2.5 mm-3.5 mm). The final results showed that the LLL of DCB-treated patients was significantly lower than the DES group (0.05 ± 0.27 mm vs. 0.40 ± 0.54 mm) during the dual antiplatelet therapy for 6 weeks (64). Several other clinical trials reported similar results confirming the effectiveness of DCB in treating macrovascular disease (63, 65, 66). Other relevant studies are shown in Table 3. However, contrasting results have been reported in the literature. For example, the meta-analysis conducted by Lin et al. showed that DCB had a higher rate of TLR in macrovascular therapy (70). The discrepancy in findings may be due to the publication bias of some current articles emphasizing the need for high-quality clinical randomized controlled trials.

**Table 3:** Clinical trials of DCBs in LVD.

**Study name**	**Country and time**	**Research design**	**Follow-up duration**	**Primary endpoint**	***p* value**	**Secondary endpoint**	***p* value**
Rosenberg et al. (67)	Germany 2019	DCB (*n* = 154)	9 months	MACE: 5.6%	–	TLR: 2.3%	–
FALCON (68)	Germany 2019	DCB (*n* = 82)	12 months	MACE: 8.0%	–	TLR: 4.9%	–
Yu et al. (63)	China 2018	DCB (*n* = 222)	10 months	MACE: 0%	–	–	–
DEBUT (69)	Finland 2017	DCB (*n* = 102) vs. BMS (*n* = 106)	9 months	MACE: 1% vs. 14%	0.00034	–	–
Lu et al. (65)	China 2017	DCB (*n* = 92)	12 months	MACE: 4.3%	–	TLR: 4.3%	–
DELUX (42)	Germany 2014	DCB (*n* = 24)	12 months	MACE: 9.4%	–	TVR: 3.1%	–
FALCON (68)	Germany 2015	DCB (*n* = 326)	12 months	MACE: 8%	–	–	–

Other abbreviations as in Table 1.

It is well-established that DCB angioplasty is associated with a high incidence of intraoperative dissection. However, it remains unclear whether intraoperative dissection affects patient outcomes. In a prospective observational study from Italy that included 156 LVD patients, 52 presented with dissection after DCB, but none underwent remedial stent implantation. After a mean of 201 days’ follow-up imaging, complete healing of the dissection was observed in 93.8% (45 patients), with no significant difference in the incidence of MACE at the 9-month follow-up among the groups (all types of entrapment/no entrapment/with entrapment). Of note, we observed late lumen enlargement in the treated segments in the dissection cohort. The study results showed self-healing of the dissection without increasing the rate of MACE (71). Consistently, an increasing body of evidence suggests that intraoperative dissection does not affect the clinical outcome of these patients (72–74).

Adopting DCB alone in the treatment of coronary macrovascular diseases has become a research hotspot. Even though recent studies have shown positive therapeutic effects, more real-world studies are warranted to corroborate the efficacy and safety of DCB application in macrovascular diseases in the future.

## 5. Application of DCB in bifurcation lesions

Bifurcation lesions account for 15 to 20% of diseases needing coronary interventions and represent a significant challenge for physicians (75). Managing bifurcation lesions is often challenging, given the high rates of branch vessel occlusion and restenosis, thrombosis of stents, and other dangerous events (76, 77). Double-stent placement is the conventional therapy for coronary bifurcation lesions but does not significantly improve the prognosis and even increases the rate of hospitalization and the incidence of MACE. Smith et al. found that the double-stent implantation could improve the in-hospital incidence of myocardial infarction and MACE (78). Moreover, the longer operative duration associated with double-stent placement increased exposure to higher X-ray doses, negatively impacting patients (79). Although the single stent placement is recommended as the first-line treatment for bifurcated lesions according to current European Bifurcation Club guidelines, single-stent implantation may still change the original anatomical structure of the bifurcation vessel and cause damage to the side branches, such as limited collateral flow, resulting in myocardial ischemia, and even complete occlusion of side branch in more severe cases (80, 81). Balloon angioplasty is an alternative for expanding the side branch vessels with the advantage of being relatively simpler without changing the original anatomical structure of the vessel. However, when only plain balloons are used in the side branch vessels after the stent is placed in the main branch, the rate of distant restenosis remains high in the main branch vessels and side branch vessels (82). Growing evidence suggests no significant reduction in the incidence of events such as MACE and myocardial infarction in patients with plain balloon angioplasty (83, 84). Excitingly, DCB represents a potential alternative to drug-eluting stents and can improve therapeutic efficacy. It can evenly deliver antiproliferative drugs to the walls of branch vessels, especially the opening of blood vessel branches, without needing additional stents (85). DCB theoretically provides complete coverage of the vascular wall without altering vascular anatomy (86, 87). DCB represents a better choice to reduce the incidence of restenosis in treating bifurcation lesions. However, DCB strategies for treating bifurcation lesions remain largely unknown. The DEBIUT study was the first to explore the efficacy of DCB in bifurcation lesions. In this study, 20 patients with bifurcation lesions underwent PCI with DCB coated with paclitaxel, followed by provisional stenting of the main branch with BMS. All operations were successful, no acute or subacute branch occlusion occurred, and only 3 months of postoperative dual antiplatelet treatment was received. At 4-month follow-up, no MACE occurred in all patients. This study provides preliminary evidence that DCB treatment of bifurcation lesions may be safe, although no follow-up imaging data were available to corroborate the effect of DCB treatment on bifurcation (88). The PEPCADV study used a similar strategy as the DEBIUT study in 28 patients with bifurcation lesions. The follow-up results of this study showed that the LLL of the main branch and the side branch at 9 months after the operation was 0.38 ± 0.46 mm and 0.21 ± 0.48 mm, and the restenosis rates were low (3.8 and 7.7%, respectively). This study provides compelling evidence that DCB is a promising treatment option (89). A similar study by Kleber et al. enrolled 64 patients and randomly divided them into DCB and plain balloon treatment groups. After 9 months of follow-up with coronary angiography, the rate of restenosis in the DCB treatment group was lower (6% vs. 26%) (90). A meta-analysis by Zheng et al. included 10 studies with a total of 934 patients, and the results showed that the short-term efficacy of the DCB group was better than the plain balloon group for the treatment of the side branch vessels of bifurcation lesions (91). Many studies have substantiated the applicability of DCB, with good efficacy in treating bifurcation lesions (92, 93).

Currently, studies on the application of DCB in side branch lesions have reported encouraging results regarding angiographic and clinical outcomes. Moreover, studies have assessed the efficacy of DCB alone in treating bifurcation lesions. Schulz et al. first documented the efficacy of DCB alone in treating bifurcation lesions, reporting an overall TLR and MACE rate of only 7.7%, highlighting that the use of DCB alone for the treatment of bifurcation lesions is safe (94). The PEPCAD-BIF trial compared the efficacy of DCB and plain balloon in treating bifurcation lesions. The results showed that the LLL was reduced in the DCB group compared to the plain old balloon angioplasty (POBA) group (0.13 mm in DCB vs. 0.51 mm in POBA, *p* = 0.01). More details are provided in Table 4 (90). Adopting DCB alone to treat bifurcation lesions represents an attractive approach. However, studies comparing the efficacy of DCB and DES in bifurcation lesions are still lacking (97), and more research is needed to confirm the efficacy of DCB in bifurcation lesions (98, 99). Besides, it should be borne in mind that bifurcation lesions often involve main and branch blood vessels and these lesions are more complex and warrant a more intricate clinical trial design.

**Table 4:** Clinical trials of DCBs in Bifurcation lesions.

**Study name**	**Country and time**	**Research design**	**Follow-up duration**	**Primary endpoint**	***p* value**	**Secondary endpoint**	***p v*alue**
**DES/BMS in MB, DCB in SB**							
DEBSIDE (92)	French 2015	DES in MB, DCB in SB (*n* = 50)	6 months	LLL in SB: −0.04 ± 0.34 mm	–	TLR: 2%	–
BIOLUX-1(74)	Australia 2015	DES in MB, DCB in SB (*n* = 35)	9 months 12 months	LLL in SB: 0.10 ± 0.43 mm	–	– TLR: 2.9%	– –
BABILON (95)	Spain 2014	BMS in MB, PCB in SB (*n* = 52) vs. DES in MB (*n* = 56)	9 months	LLL: in MB:0.31 ± 0.48 mm vs. 0.16 ± 0.38 mm In SB: −0.03 ± 0.51 mm vs. 0.04 ± 0.76 mm	0.15 0.983	MACE: 17.3% vs. 7.1% TLR: 15.4% vs. 3.6%	0.105 0.045
DCB Bifurcation Study (96)	Spain 2012	DES in MB, PCB in SB (*n* = 50) vs. DES in MB, POBA (*n* = 50) in SB	12 months	LLL: 0.09 ± 0.4 mm vs. 0. 40 ± 0.5 mm	0.01	MACE: 11% vs. 24% TLR: 12% vs. 22%	0.11 0.16
PEPCAD V (89)	Germany 2010	BMS in MB, DCB in SB (*n* = 28)	9 months	LLL in SB:0.21 ± 0.48 mm	–	MLD in SB: 1.7 ± 0.44 mm	–
DEBIUT (88)	The Netherlands 2008	BMS in MB, DCB in SB (*n* = 20)	4 months	MACE: 0%	–	–	–
**DCB-Only in SB**							
PEPCAD-BIF (90)	Germany 2016	DCB (*n* = 32) vs. POBA (*n* = 32)	9 months	LLL: 0.13 mm vs. 0.51 mm	0.013	Restenosis rate: 6% vs. 26	0.045
Schulz (94)	Germany 2014	DCB-Only in SB (*n* = 39)	4 months	MACE:7.7%7%	–	–	–

MB, main branch; SB, side branch; other abbreviations as in Table 1.

## 6. Application of DCB in chronic total occlusion

Chronic Total Occlusion (CTO) accounts for 16 to 52% of coronary artery disease and is defined by two main criteria: (1) no antegrade blood flow through lesions; (2) the presumed or diagnosed duration time ≥ 3 months (100). In CTO, patients with long-term chronic ischemia often have good collateral circulation; however, these collateral vessels maintain cardiac function in only about 5% of patients, and more than 3 quarters of CTO patients still have ischemic zones in their hearts (101, 102). It is widely thought that aggressive revascularization could reduce ischemia, all-cause mortality and the rate of nonfatal myocardial infarction in patients with coronary CTO (101). Moreover, the clinical outcomes of coronary intervention in CTO could be better than pharmacological treatment (103). According to the 2018 ESC/EACS guidelines for myocardial revascularization, CTOs should be opened when there is still refractory angina pectoris after drug treatment or with evidence of extensive myocardial ischemia and occlusion of blood vessels, and PCI application is recommended for CTO patients (level of evidence IIA/B) (44).

The implantation of DES improves the prognosis of CTO patients but still faces problems such as poor stent coverage, late stent thrombosis and a high restenosis rate. Yang et al. (104) reported that 14.21% of CTO patients treated with stent therapy experienced restenosis, higher than reported by Suttorp et al. (14%) (105) and Valenti et al. (12.5%) (106), which may be attributed to foreign body implantation during stent therapy. Therefore, DCB alone may be a suitable choice for treating CTO disease. A German group published the first multicenter study about DCB treatment for CTO patients in 2016. Good outcomes were reported with a vascular recanalization rate of 79.4% (n = 27) with only one case of restenosis but no myocardial infarction or death. Although the small sample size limited the reliability of these findings, the study highlighted the applicability of DCB in the treatment of CTO (107). A Korean study using DCB alone to treat CTO demonstrated that the LLL in the DCB group was 0.03 mm, better than the DES group (0.15–0.20 mm) (108). Meanwhile, several case reports listed in Table 5 concluded that DCB is a feasible method for treating CTO lesions (110, 111).

**Table 5:** Clinical trials of DCBs in CTO.

**Study name**	**Country and time**	**Research design**	**Follow-up duration**	**Primary endpoint**	***p* value**	**Secondary endpoint**	***p* value**
Shin (108)	South Korea 2021	DCB (*n* = 84)	24 months	MACE: 16.7%	–		–
Köln et al. (107)	Germany 2016	DCB (*n* = 34)	4 weeks	Recanalization rate: 79.4% (n = 27)	–	Restenosis rate: 11.8% (*n* = 4)	–
PEPCAD CTO (109)	Germany 2012	DCB + BMS (*n* = 48) vs. DES (*n* = 48)	6 months 12 months	LLL: 0.33 ± 0.69 mm vs. 0.26 ± 0.70 mm	0.65	Restenosis rate: 27.7 vs. 20.8% MACE: 14.6% vs.18.8%	0.44 0.58

Other abbreviations as in Table 1.

Previous studies have demonstrated that the number/length of stents is associated with various adverse events (112, 113). Interestingly, long stent implantation affects vasomotor activity and promotes neo-atherosclerosis (114, 115). The HYPER study showed that the combination therapy of DCB and DES in diffuse lesions could reduce the total stent length applied in the vessel and decrease the incidence of MACE compared with the DES alone group (20.8% vs. 22.7%). It was concluded that DCB is an effective choice of combination therapy to improve the prognosis of patients (116). PEPCAD-CTO compared DES and DCB combined with bare metal stents for treating CTO patients. Although the study found no difference in clinical endpoints within 12 months, the group treated with DCB combined with bare metal stents required a shorter dual antiplatelet regimen. Besides, no late in-stent thrombosis occurred in the combination therapy group, while one late in-stent thrombosis occurred in the DES group, highlighting the superior long-term clinical outcomes of DCB over DES (109). DCB combined with stent implantation is a feasible and well-tolerated treatment in CTO lesions which is likely to benefit the prognosis of patients.

During clinical practice, CTO remains one of the most challenging types of coronary artery disease. Patients with CTO often involve multiple lesions or are combined with other complications and a high risk of bleeding (117). The usage of DCB shortens stent implantation and benefits most patients in terms of prognosis, highlighting the potential role of DCB to act as an adjuvant or definite treatment for CTO patients and requiring the use of other techniques, which makes it challenging for the physician (118).

## 7. Summary and prospect

DCB brings many advantages during PCI therapy of coronary disease, circumventing the need for foreign body implantation. Compared with stent placement, DCB can reduce the risk of in-stent restenosis and late in-stent thrombosis, and even late lumen enlargement can occur (119, 120). DCB has been suggested for the therapy of small vessel disease and ISR, with substantial evidence confirming its efficacy and safety. More research explored the application of DCB for new indications, such as bifurcation lesions, macrovascular lesions, chronic total occlusive lesions, etc. Interestingly, these studies also showed positive results highlighting that DCB has huge prospects for a wider range of applications. However, there are still some shortcomings in currently published studies, such as small sample sizes and the lack of real-world assessment. The results of multicenter studies with large samples and long-term and standardized trial registration design are highly anticipated to provide more clinical evidence for DCB in complex coronary artery disease treatment.

DCB enables an even drug distribution at the lesion. At the same time, the retention and efficacy of locally administered drugs are determined by vessel anatomy and lesion morphology (121). In lesions, calcium, lipids, fibers, and hemorrhagic entities affect drug uptake and device implantation. In lipid lesions, which are often accompanied by diffuse intimal thickening and affect drug binding, sirolimus-based delivery is more effective due to its lower sensitivity to lipid entities (122). The calcified plaques affect drug permeability and retention in the coronary artery and reduce vascular mechanical compliance (123). In addition, using DCB to dilate hard lesions can cause dangerous conditions such as dissection. Studies have shown that good pre-dilation with cutting or scoring balloons prior to implantation can achieve better therapeutic outcomes (124). Fibrotic lesions in blood vessels tend to be unstable, and surface rigidity hinders acute coating transfer by DCB. Accordingly, DCB is not a better option than DES. Interestingly, the diverse results from heterogeneous lesion phenotypes could be rectified through vessel preparation or testing instruments; however, definitive large-scale clinical outcome results remain to be established in this arena.

With the development of new drugs for coating, a new opportunity and era will emerge, leading to increased use of DCB in treating coronary artery disease, hopefully alleviating some of the concerns raised by DES (125). At the same time, significant emphasis has been placed on novel methods, including DCB combined with bioabsorbable stents or directional coronary atherectomy (126). Ongoing real-world research can also help us with the optimal use of DCB in this patient population (127). Clinical studies are ongoing to evaluate the application scenarios. Nicola S Vos et al. assessed the efficacy and safety of DCB versus DES in primary percutaneous coronary intervention for ST-segment elevation myocardial infarction (STEMI) and found that the effect of DCB was not inferior to DES (128). Similar studies are in progress. In addition to coronary arteries, its application in other areas is being studied, such as peripheral artery disease (129), urethral stricture, vertebral artery stenosis, and intracranial atherosclerosis. It is widely believed that with the design of more clinical trials and the development of novel drugs and corresponding technologies, the clinical application of DCB in coronary artery disease will gain broader acceptance.

## Author contributions

LW, XL, and LL contributed to the study conception, design, and drafted the article. HW, TL, and CW revised the manuscript for intellectual content and approved the final version to be published. All authors contributed to the article and approved the submitted version.

## Funding

This work was supported by the Natural Science Foundation of Shaanxi Province (2022JQ-921).

## Conflict of interest

The authors declare that the research was conducted in the absence of any commercial or financial relationships that could be construed as a potential conflict of interest.

## Publisher’s note

All claims expressed in this article are solely those of the authors and do not necessarily represent those of their affiliated organizations, or those of the publisher, the editors and the reviewers. Any product that may be evaluated in this article, or claim that may be made by its manufacturer, is not guaranteed or endorsed by the publisher.
